# Performance Evaluation of an Entropy-Based Structural Health Monitoring System Utilizing Composite Multiscale Cross-Sample Entropy

**DOI:** 10.3390/e21010041

**Published:** 2019-01-09

**Authors:** Tzu-Kang Lin, Yi-Hsiu Chien

**Affiliations:** Department of Civil Engineering, National Chiao Tung University, Hsinchu 30010, Taiwan

**Keywords:** structural health monitoring, multi-scale, composite cross-sample entropy

## Abstract

The aim of this study was to develop an entropy-based structural health monitoring system for solving the problem of unstable entropy values observed when multiscale cross-sample entropy (MSCE) is employed to assess damage in real structures. Composite MSCE was utilized to enhance the reliability of entropy values on every scale. Additionally, the first mode of a structure was extracted using ensemble empirical mode decomposition to conduct entropy analysis and evaluate the accuracy of damage assessment. A seven-story model was created to validate the efficiency of the proposed method and the damage index. Subsequently, an experiment was conducted on a seven-story steel benchmark structure including 15 damaged cases to compare the numerical and experimental models. A confusion matrix was applied to classify the results and evaluate the performance over three indices: accuracy, precision, and recall. The results revealed the feasibility of the modified structural health monitoring system and demonstrated its potential in the field of long-term monitoring.

## 1. Introduction

Over the preceding few decades, structural health monitoring (SHM) techniques have been developed for early damage detection in various engineering fields. Traditional SHM techniques such as visual inspection tend to be time consuming and rely heavily on manpower; therefore, these techniques have low generalizability. Recently, novel SHM methods based on signal processing techniques have been proposed for analyzing measured responses in succession. Dynamic monitoring entails measuring the displacement, velocity or acceleration signals of structures to obtain time–frequency characteristics.

In 1999, Wahab and De Roeck [1] utilized the change in modal curvature between the undamaged and damaged conditions of simply supported and continuous beams to detect damage in prestressed concrete beams. In 2000, Maeck [2] identified the location and degree of damage in reinforced concrete (RC) beams by conducting dynamic stiffness analysis. In 2003, Chang [3] summarized the limitations and applications of vibration-based SHM methods. The impact of measuring noise, environmental and damage on the sensitivity of the damage detection was then analyzed [4]. In 2016, Amezquita-Sanchez et al. summarized the current signal processing techniques for vibration-based SHM and point out its advantages and disadvantages [5]. Opoka et al. then applied the root mean square deviation (RMSD) estimator to detect the damage by comparing the averaged frequency spectrum [6]. In 2018, Soman et al. proposed a two-step methodology based on frequency spectrum for the damage detection and localization [7]. This method used the change of the frequency response function (FRF) spectrum to distinguish the healthy and the damaged tripod structure. However, complex structural behavior is difficult to capture because of incomplete measurements and the randomness of ambient vibration [8,9].

Information theory can be applied to study the quantification, storage, and communication of information. In 1948, Shannon [10] proposed Shannon entropy as a measure of uncertainty in the outcomes of random processes. Subsequently, Kolmogorov [11] defined the notion of entropy for a new class of dynamical systems, and Sinai [12] introduced a definition of entropy that can be applied to all dynamical systems. The Kolmogorov–Sinai entropy (KS entropy) has been used to measure the complexity of time series in D-dimensional dynamic systems and is crucial in ergodic theory. Subsequent studies have demonstrated that KS entropy results are affected by various levels of noise during analysis of experimental data [13].

In 1991, Pincus [14] modified KS entropy and named the modified version approximate entropy (ApEn). ApEn was initially utilized to analyze medical data such as heart rate and served as a regularity statistic for quantifying the unpredictability of fluctuations over a time series. An and Ou [15] proposed the mean curvature difference method based on ApEn theory and successfully used the proposed method to locate damage in shear frame structures. In 2000, Richman and Moorman [16] further modified ApEn to develop sample entropy (SampEn). SampEn has two advantages over ApEn: data length independence and higher relative consistency under different parameters. Lake et al. [17] used SampEn to investigate neonatal heart rate variability and validate the characteristics of SampEn.

In 2002, Costa et al. [18] proposed multiscale entropy (MSE) to solve the problem of distinguishing between healthy individuals, patients with congestive heart failure, and patients with erratic cardiac arrhythmia on a single time scale. Consequently, the coarse-graining procedure was proposed to obtain more reliable results during entropy calculation [19]; the results demonstrated that the time-series structure exhibited complexity loss under pathologic conditions on a multiple time scale. MSE has been used extensively in not only medicine but also mechanical engineering and finance. MSE and an adaptive neuro-fuzzy inference system were utilized to distinguish between fault damage categories and identify levels of fault severity [20]. Xia and Shang [21] applied MSE to investigate degrees of self-match and measure complexity in the American, European, and Asian stock markets.

Cross-ApEn was introduced by Pincus and Singer [22] in 1996 for analyzing the degree of asynchrony between two related time series. Furthermore, Richman and Moorman [16] proposed Cross-SampEn for measuring the asynchrony and dissimilarity between two distinct time series. The results indicated that Cross-SampEn was a more consistent measure of joint synchrony between pairs of clinical cardiovascular time series. In 2013, Fabris et al. [23] extended applications of SampEn and Cross-SampEn to analyze electroglottogram and microphone signals. Healthy patients and patients with throat or voice disorders could be identified by evaluating the degree of asynchrony between two time series.

Although the MSE algorithm has been successfully applied in multiple fields, a problem has emerged in practical applications: the statistical reliability of SampEn decreases as the corresponding time scale increases. Therefore, in 2013, Wu et al. [24] introduced composite MSE (CMSE) to overcome the problem. To validate the applicability of CMSE to real data, the acceleration signals of bearing faults were analyzed to demonstrate that CMSE provided more accurate entropy values than did MSE. In 2016, Yin et al. [25] proposed composite multiscale cross-sample entropy (CMSCE) to address accuracy concerns regarding the MSCE method. Subsequently, CMSCE was applied to analyze the asynchrony between financial time series, which included six stock indices from multiple regions.

**Figure 1:**
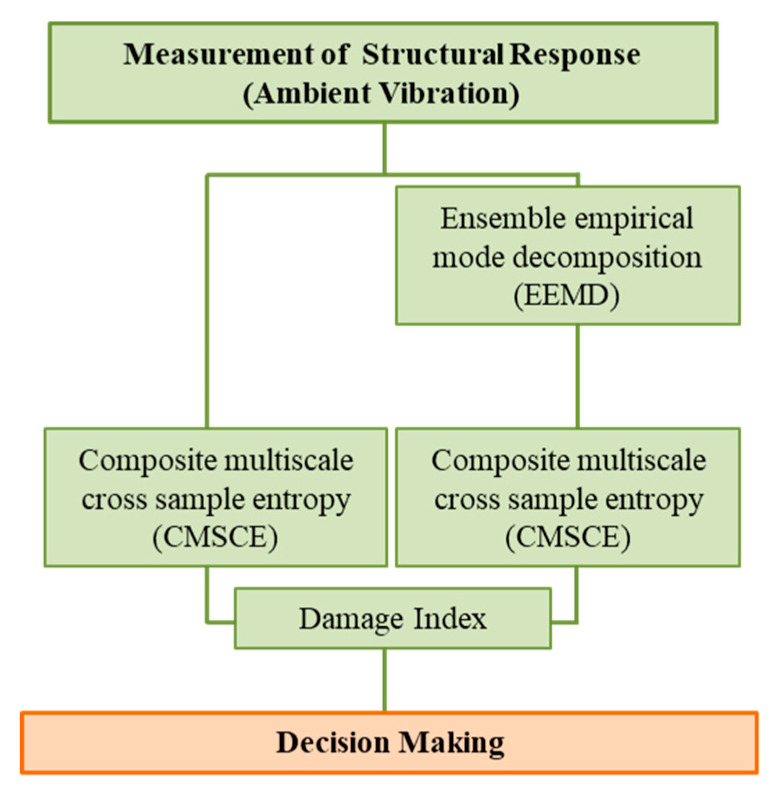
Flowchart of the study.

The aim of this study was to develop an entropy-based SHM system for solving the problem of unstable entropy values observed when multiscale cross-sample entropy (MSCE) is employed to assess damage in laboratory-scale structure. The flowchart of the study procedures is shown in Figure 1. The proposed system was validated by simulating the ambient vibration response of a seven-story model and conducting a steel structure experiment. The health condition was first detected by the ambient vibration response through CMSCE. Moreover, as most of the practical damages were observed on the lower part of a structure under earthquake excitation, monitoring the change of the first fundamental mode frequency is an alternative way for rapid screening. Therefore, ensemble empirical mode decomposition (EEMD) was attempted to extract the first mode of structural response to evaluate the feasibility of detecting damage according to only the first mode [26]. A previously proposed damage index (DI) was used to efficiently quantify damage, and the decision of damage location and condition was made.

## 2. Methodology

### 2.1. Cross-SampEn Method

Cross-SampEn is used to evaluate the degree of dissimilarity between two time series from the same system. The procedure of Cross-SampEn is similar to that of SampEn and can be summarized as follows [16]. First, consider two individual time series $\left\{X_{i}\right\}{\;=\;\{x}_{1}{,\;\ldots ,\;x}_{i}{,\;\ldots ,\;x}_{N}\}$ and $\left\{Y_{j}\right\}{\;=\;\{y}_{1}{,\;\ldots ,\;y}_{j}{,\;\ldots ,\;y}_{N}\}$ with length *N*. Two signals are divided into templates of length m: $u_{m}\left(i\right){\;=\;\{x}_{i}{,\;x}_{i+1}{,\;\ldots ,\;x}_{i+m-1}\},\;1\;\leq \;i\;\leq \;N\;–\;m\;+\;1$ and $v_{m}\left(j\right){\;=\;\{y}_{j}{,\;y}_{j+1}{,\;\ldots ,\;y}_{j+m-1}\},\;1\;\leq \;j\;\leq \;N\;–\;m\;+\;1$. Therefore, two template spaces $T_{x}$ and $T_{y}$ are presented as follows:

(1) $$T_{x}\;=\;\left[\begin{array}{c} \begin{array}{cccc} x_{1} & x_{2} & \cdots & x_{m}\\ x_{2} & x_{3} & \ldots & x_{m-1}\\ \vdots & \vdots & \ddots & \vdots \\ x_{N-m+1} & x_{N-m+2} & \cdots & x_{N} \end{array} \end{array}\right]\;T_{y}\;=\;\left[\begin{array}{c} \begin{array}{cccc} y_{1} & y_{2} & \cdots & y_{m}\\ y_{2} & y_{3} & \ldots & y_{m-1}\\ \vdots & \vdots & \ddots & \vdots \\ y_{N-m+1} & y_{N-m+2} & \cdots & y_{N} \end{array} \end{array}\right]$$

The number of similarities between $u_{m}\left(i\right)$ and $v_{m}\left(j\right)$ is defined as $n_{i}^{m}\left(r\right)$, which can be expressed as follows:

(2) $$n_{i}^{m}\left(r\right)\;=\sum_{j=1}^{N-m}d\left[u_{m}\left(i\right){,\;v}_{m}\left(j\right)\right]$$

where $d\left[u_{m}\left(i\right){,\;v}_{m}\left(j\right)\right]$ is the maximum distance between two templates *i* and *j.* Under the condition that the maximum distance is within *r*, which is a predetermined threshold, $n_{i}^{m}\left(r\right)$ can be calculated.

(3) $$d\left[u_{m}\left(i\right){,\;v}_{m}\left(j\right)\right]\;=\;\max\{|x\left(i+k\right)-y\left(j+k\right)|:\;0\;\leq \;k\;\leq \;m\;-\;1\}$$

(4) $$d\left[u_{m}\left(i\right),\;v_{m}\left(j\right)\right]\;\leq \;r,\;1\;\leq \;j\;\leq \;N\;-\;m$$

The similarity probability of a template is calculated using the following equation:

(5) $$U_{i}^{m}\left(r\right)\left(v\|u\right)\;=\;\frac{n^{m}\left(r\right)}{\left(N\;-\;m\right)}$$

Subsequently, the average probability of similarity for template *m* is calculated as follows:

(6) $$U^{m}\left(r\right)\left(v\|u\right)\;=\;\frac{1}{(N\;-\;m)}\sum_{i=1}^{N-m}U_{i}^{m}\left(r\right)\left(v\|u\right)$$

where $U^{m}\left(r\right)\left(v\|u\right)$ is the degree of asynchrony between the two template spaces, which are segmented with length *m*. Next, new template spaces $T_{x}$ and $T_{y}$ are created with different template lengths *m + 1*, and then the procedure for calculating the probability of similarity is repeated to obtain the average probability of similarity $U^{m+1}\left(r\right)\left(v\|u\right)$. Cross-SampEn is expressed as follows:

(7) $${CS}_{E}\left(m,\;r,\;N\right)\;=-\ln\left\{\frac{U^{m+1}\left(r\right)\left(v\|u\right)}{U^{m}\left(r\right)\left(v\|u\right)}\right\}$$

### 2.2. MSE Method

Compared with sample entropy values on a single time scale, MSE is capable of extracting more information from a time series [12,13]. New time series are constructed by performing coarse graining. The original time series is divided into nonoverlapping windows with time scale *τ* from 1 to *N*. A new time series $\left\{y_{j}^{\left(\tau \right)}\right\}$ is constructed by taking the arithmetic mean of each window. The equation is as follows:

(8) yj(τ) = 1τ∑i= (j −1)τ+1jτxi, 1 ≤ j ≤ Nτ

The length of each created coarse-grained time series is *N*/τ. After the coarse-graining procedure, SampEn is conducted for each coarse-grained time series $\left\{y_{j}^{\left(\tau \right)}\right\}$. Then, the obtained entropy values are considered the multiscale sample entropy of the time series and can be plotted as a function of the time scale factor $\left(f\left(\tau \right){\;=\;S}_{E}\right).$ The MSE error grows as the length of the time series decreases, particularly when the response of a real structure is being measured.

### 2.3. CMSE Method

CMSE was proposed by Wu et al. [24] for improving the accuracy of MSE. CMSE defines the *k*th coarse-grained time series for a scale factor of τ. Therefore, each coarse-grained time series can be obtained using the following equation:

(9) yk,j(τ) = 1τ∑i=(j −1)τ+kjτ+k−1xi, 1 ≤ j ≤ Nτ, 1≤k≤τ

The CMSE algorithm produces τ coarse-grained time series at scale factor τ. Subsequently, SampEn values for all coarse-grained time series are calculated, and the CMSE value is defined as the mean of τ SampEn values, as shown in Equation (10):

(10) $$\mathrm{CMSE}\left(x,\tau ,m,r\right)=\frac{1}{\tau }\sum_{k=1}^{\tau }SampEn\left(y_{k}^{\left(\tau \right)},m,r\right)$$

Based on the concept of CMSE, CMSCE proposed by Yin et al. [25] is defined as follows:

(11) $$\mathrm{CMSCE}\left(x,y,\tau ,m,r\right)=\frac{1}{\tau }\sum_{k=1}^{\tau }SampEn\left(x_{k}^{\left(\tau \right)},y_{k}^{\left(\tau \right)},m,r\right)$$

### 2.4. DI Measure

Inspired by a series of biomedical studies that have used the area of the MSE curve as an index for quantifying complexity [27,28,29], the DI was proposed as a measure for rapidly and efficiently diagnosing the floor damage of a structure. Signals are considered more complex when their entropy values are higher than those of other signals. For a structure with *F* floors, the CMSCE curves under undamaged and damaged conditions can be respectively expressed as follows:

(12) $${\mathrm{CMSCE}}_{\mathrm{undamaged}}\;=\;\left\{\begin{array}{c} H_{1}\\ H_{2}\\ \vdots \\ H_{F} \end{array}\right\}{\;\mathrm{CMSCE}}_{\mathrm{damaged}\;}=\left\{\begin{array}{c} D_{1}\\ D_{2}\\ \vdots \\ D_{F} \end{array}\right\}$$

where *H* and *D* represent the undamaged and damaged conditions, respectively. The subscript *F* indicates the analyzed floor; for example, $H_{1}$ is the Cross-SampEn of signals between the foundation and the first floor under the undamaged condition. Moreover, $H_{1}$ can be expressed as matrices: $H_{1}\;=\;\left\{{CS}_{E}^{H_{11}}{,\;CS}_{E\;}^{H_{12}}{,\;CS}_{E\;}^{H_{13}},\;\cdots {,\;CS}_{E\;}^{H_{1\tau }}\right\}$, where ${CS}_{E\;}^{H_{1\tau }}$ represents the Cross-SampEn of the first floor at scale factor τ. Hence, the general expression of Cross-SampEn on floor *F* is as follows:

(13) $$D_{F}\;=\;\left\{{CS}_{E}^{D_{F1}}{,\;CS}_{E\;}^{D_{F2}}{,\;CS}_{E\;}^{D_{F3}},\;\cdots {,\;CS}_{E\;}^{D_{F\tau }}\right\}$$

The DI is evaluated by calculating the differences between the areas of CMSCE curves from scale 1 to τ. The DI of floor *F* can be expressed as follows:

(14) $$DI_{F}=\sum_{q=1}^{\tau }\left(CS_{E}^{D_{Fq}}-CS_{E}^{H_{Fq}}\right)$$

A positive DI indicates that the floor is damaged, whereas a negative DI indicates that the floor is undamaged. Based on results from previous studies, the DI performs to a satisfactory degree in numerical models that contain 10–20% noise. Therefore, in this study, the DI is utilized to identify the damaged floor in the numerical model and experiment.

## 3. Feasibility Assessment

### 3.1. Comparison of MSCE and CMSCE

**Figure 2:**
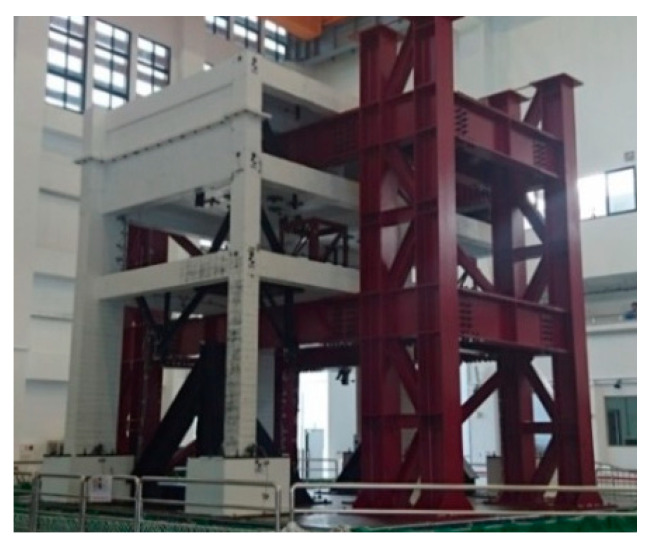
Three-story RC structure.

**Figure 3:**
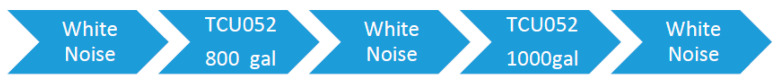
Shaking table test procedure.

According to the experience of applying MSCE for SHM, the optimized template length *m* was set as 4 based on the characteristics of recorded time series [30]. However, inevitable fluctuation was occasionally observed on the calculated entropy curve, which makes the diagnosis of damage condition and location difficult. To verify the ability of CMSCE to provide accurate entropy values subject to a long template length, experimental data obtained from a shaking table test were first analyzed to compare the performance of MSCE and CMSCE in this section. The shaking table test with alternating spells of white noise and TCU052 earthquake was conducted on a two-bay three-story reinforced concrete (RC) structure, which is shown in Figure 2. The procedure is shown in Figure 3. The acceleration of each floor was measured for SHM. The entropy-based monitoring system was based on ambient vibration, which was simulated by white noise during the shaking table test. The acceleration response of the first white noise iteration was used to represent the healthy status of the RC structure; the acceleration responses of the second and third white noise iterations were considered as unknown conditions after 800 and 1000 gal earthquakes.

**Figure 4:**
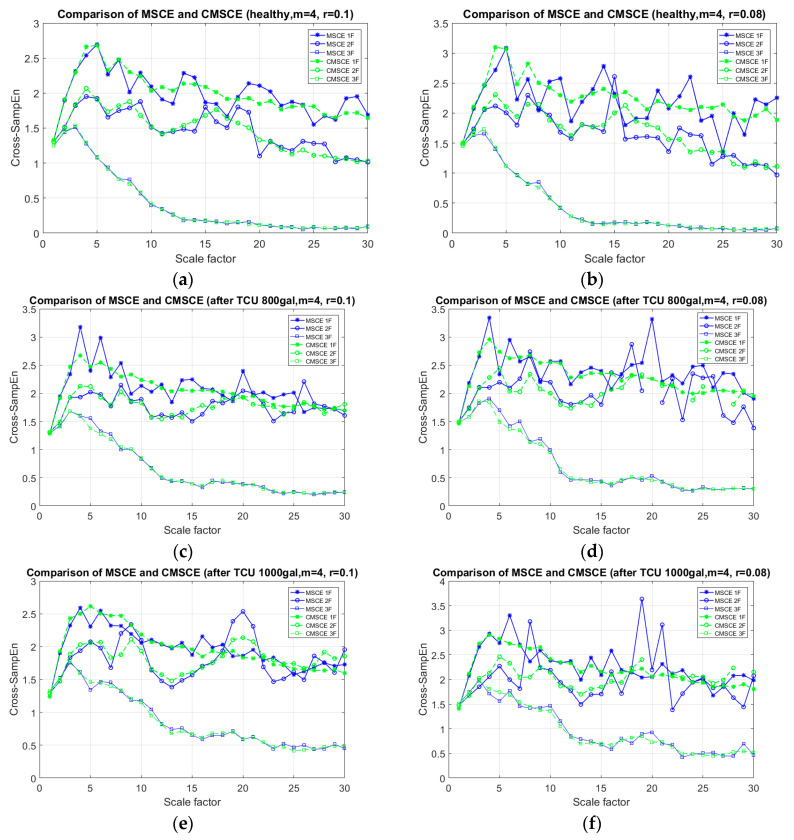
Comparison diagram of MSCE and CMSCE for: (**a**) healthy condition with *m* = 4, *r* = 0.1; (**b**) healthy condition with *m* = 4, *r* = 0.08; (**c**) after TCU 800 gal with *m* = 4, *r* = 0.1; (**d**) after TCU 800 gal *m* = 4, *r* = 0.08; (**e**) after TCU 1000 gal with *m* = 4, *r* = 0.1; (**f**) after TCU 1000 gal with *m* = 4, *r* = 0.08.

The sampling rate was 200 Hz for 120 s as the shaking table was running the white noise signal. The recorded time series were analyzed using MSCE and CMSCE with identical parameters: template length *m* = 4, threshold *r* = 0.1 × standard deviation (SD), and 0.08 × SD. Figure 4 shows the results of MSCE and CMSCE under various conditions to illustrate the necessity of using CMSCE for on-site monitoring.

Diagrams obtained for the healthy condition are presented in Figure 4a,b; the diagram obtained when *m* = 4 and *r* = 0.1 is on the left, and that obtained when *m* = 4 and *r* = 0.08 is on the right. The figure shows that the results of MSCE were fluctuated markedly compared with those of CMSCE, especially on the low floor curves. Similarly, an unstable trend of MSCE was observed (Figure 4c–f), whereas CMSCE provided a more stable and reliable post-earthquake trend. The entropy values of MSCE became more undulant as the threshold *r* decreased. Based on the results of the RC structural experiment, CMSCE was chosen to mitigate the fluctuation concern of MSCE encountered during the analysis of the data, which contained a certain level of noise.

### 3.2. Numerical Simulation

**Figure 5:**
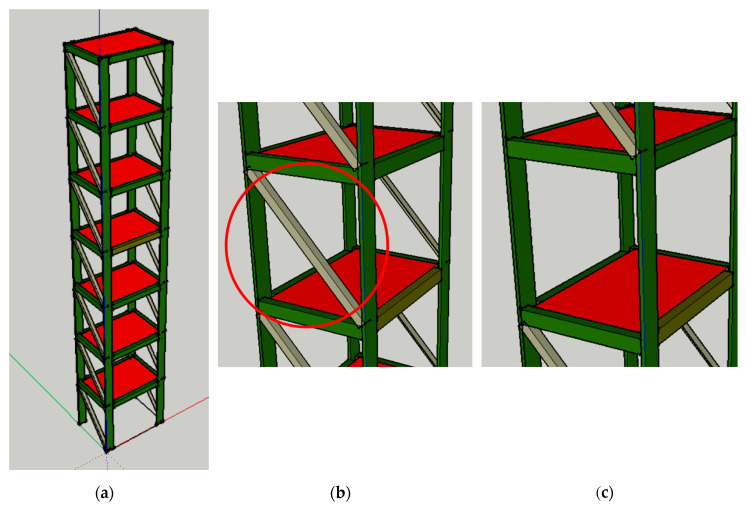
(**a**) View of the numerical model. (**b**) The undamaged scenario. (**c**) The damaged scenario.

After the comparison of the MSCE and CMSCE algorithms through the shaking table test, the advantage of CMSCE for SHM was validated. Subsequently, a numerical simulation was executed to assess the feasibility of using CMSCE on more complex structures. SAP2000 software was used to construct and analyze a seven-story model for numerical simulation. Regarding geometric properties, the model was a steel structure with a yield strength $F_{y}\;\mathrm{of}\;2500\;\mathrm{kg}/{\mathrm{cm}}^{2}$. The height of each story was 1.06 m, and the floor widths on the x- and y-axes were 1.32m and 0.92 m, respectively. The beam was a steel plate measuring 100 mm × 70 mm. The column was a steel plate measuring 25 mm × 150 mm. Steel bracings were set up on the y-axis and selected as L-shaped steel angles of 65 mm × 65 mm × 6 mm. Moreover, an additional mass of 500 kg was added to each floor to simulate real structural behavior. Structural damage was simulated by removing the bracings symmetrically. The SAP2000 model and damage scenario are shown in Figure 5.

**Figure 6:**
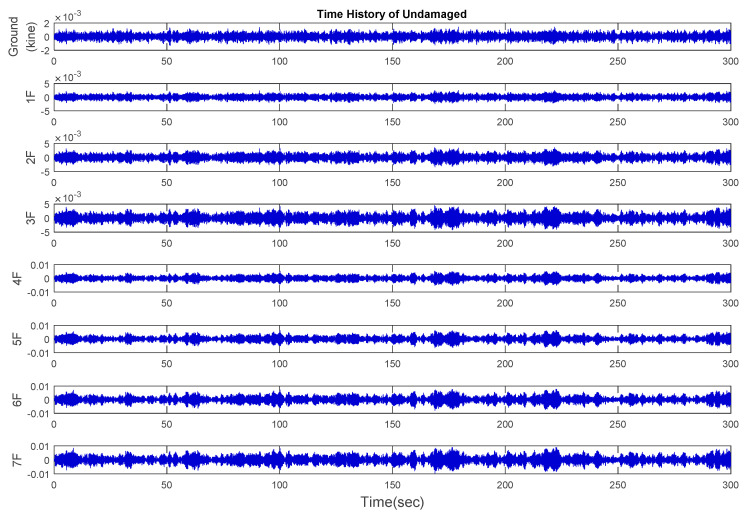
The time history of the velocity response of the undamaged case.

Time history analysis was performed using a white noise signal of 1 MW power as the input acceleration to simulate the response of the structure under ambient vibration. The sampling rate was 200 Hz for 300 s. After the database had been created on the basis of the time history analysis of each damaged case, the velocity response data, which is relatively sensitive for ambient vibration, were extracted from the center of each floor on the y-axis [31]. The responses of velocity signals in the undamaged case are shown in Figure 6. All damaged cases and the modal analysis results are presented in Table 1.

EEMD was utilized to extract the first mode of structural response for evaluating SHM performance based on the first mode. In the simulation, the results obtained with and without EEMD were displayed to evaluate the effectiveness of using the first mode to detect damage. EEMD can efficiently decompose signals into several intrinsic mode functions (IMFs) with a trend, and this is because the decomposition is based on the local characteristic time scale of the data [20,25]. The first mode of the model appeared in IMF4, and the frequencies are listed in Table 1. Subsequently, the extracted IMF4 and the original velocity response were analyzed separately to assess the damage location by using CMSCE.

### 3.3. Numerical Simulation Results

#### 3.3.1. Damage Detection from the Original Velocity Response

In every damaged case, the velocity signals of two vertically adjacent floors were processed through CMSCE after the coarse-graining procedure to evaluate the degree of dissimilarity between floors. According to previous studies that have discussed detection accuracy under various parameter combinations, parameters such as the template length *m*, threshold *r*, and signal length *N* were optimized as 4, 0.08 × SD of the time series, and 20,000 points, respectively. Additionally, the required data length recommended by Gow et al. [32] was set between 14^m^ and 23^m^ points in the final MSE scale analysis. Therefore, the DI was conservatively calculated to time scale 10 (τ = 10) for the better results. After analysis of the undamaged case and 14 damaged cases, the entropy curves for the damaged cases were compared with those for the undamaged case by calculating the DI. When a floor is damaged, the DI is positive, indicating that the signal complexity increases because of a loss of story stiffness. By contrast, a negative DI indicates a healthy floor.

**Figure 7:**
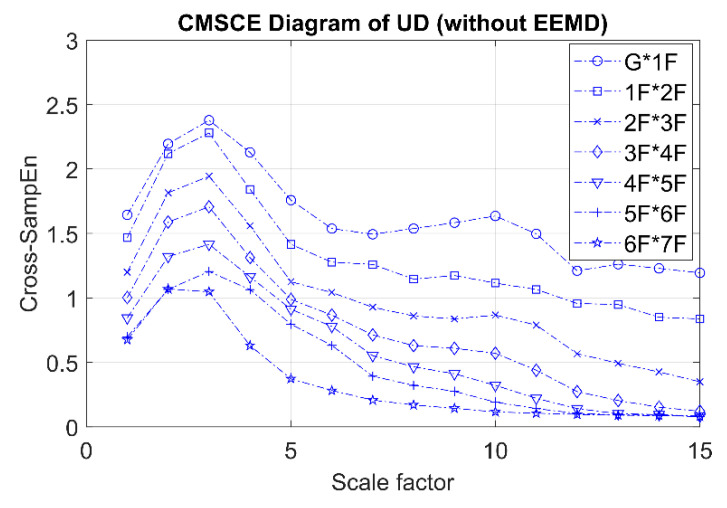
CMSCE diagram of undamaged case.

**Figure 8:**
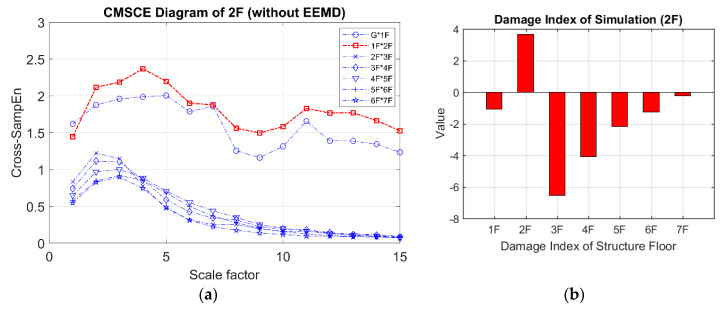
(**a**) CMSCE diagram of damage on the second floor (2F); (**b**) Damage index of damage on the second floor.

Figure 7 presents the CMSCE curves for the undamaged case. In the figure, G*1F denotes the curve for the first floor and 1F*2F denotes the curve for the second floor; the curves for the remaining floors follow similar designations. Curve G*1F had the highest entropy values, indicating high complexity and low similarity; other curves ranked further down in the order, showing a decline in complexity between the two vertically adjacent floors. The case of single-floor damage is illustrated in Figure 8. The curve for the second floor was the highest among all curves. Furthermore, Figure 8a shows a substantial gap between the curve for the second floor and the remaining curves; an increase in complexity due to a loss of story stiffness could be clearly observed. According to the DI results shown in Figure 8b, the positive DI indicated that the damage occurred on the second floor.

**Figure 9:**
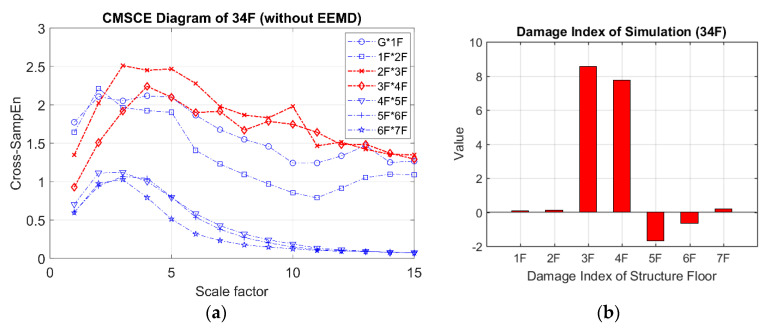
(**a**) CMSCE diagram of damage on the third and the fourth floor (34F); (**b**) Damage index of damage on the third and the fourth floor.

The results obtained for damage on the third and fourth floors are presented in Figure 9. Figure 9a shows that the curves for the third and fourth floors were higher than that for the undamaged case. Additionally, the curves for the third and fourth floors maintained higher positions than did those for the other floors at scale 5 to 15. In Figure 9b, the bars representing the third and fourth floors exhibit significantly positive values. Although extremely low positive values were observed for the first, second, and seventh floors, these could be disregarded because they were within the error tolerance range because the curves for the undamaged case maintained an almost identical level of complexity, resulting in low negative or zero-approaching values.

**Figure 10:**
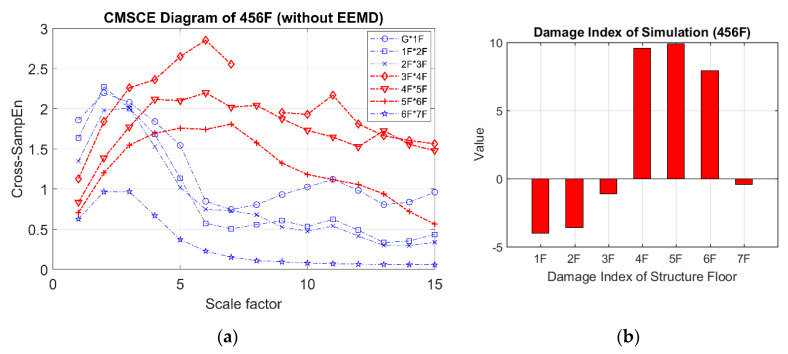
(**a**) CMSCE diagram of damage from the fourth to the sixth floor (456F); (**b**) Damage index of damage from the fourth to the sixth floor.

In the case of three-story damage, Figure 10a illustrates the CMSCE curves for damage on floors ranging from the fourth floor to the sixth floor, revealing that the curves for the fourth floor to the seventh floor climbed up at scale 5 to 15. Compared with the curve for the undamaged case, the curves for the fourth floor to the seventh floor increased, indicating an increase in complexity. Hence, the evident increase could be quantified through the DI analysis, as depicted in Figure 10b. The fourth floor to the sixth floor had positive values, indicating the presence of damage; however, few undefined entropies occurred on the fourth floor.

**Figure 11:**
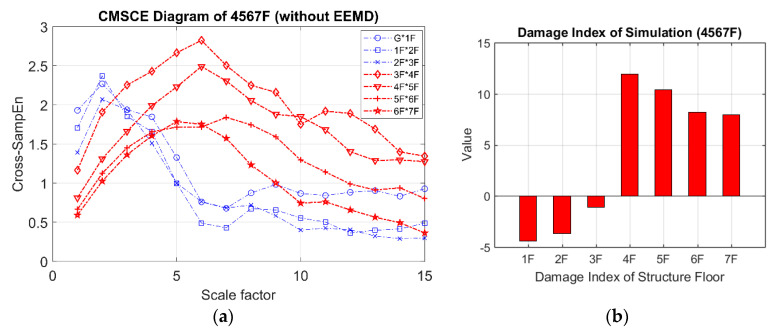
(**a**) CMSCE diagram of damage from the fourth to the seventh floor (4567F); (**b**) Damage index of damage from the fourth to the seventh floor.

Figure 11 illustrates the curves for damage on floors ranging from the fourth floor to the seventh floor. These curves were markedly higher than those for the undamaged case at scale 5 to 15. The peak values of the damaged curves shifted slightly from scale 3 to 6, indicating that maximum complexity occurred after the coarse-graining procedure. The DI diagram for multistory damage is shown in Figure 11b; the positive indices for the fourth floor to the seventh floor can easily be identified as indicating damage.

#### 3.3.2. Damage Detection from the Extracted First Mode Time Series (IMF4)

A study on sample entropy noted that signals with higher frequencies may have unpredictable effects on the calculations of SampEn values [32]; however, ambient noise usually contains a high percentage of high-frequency noise in real applications, and most of the practical structural damage mainly causes a frequency change on the first fundamental mode. Hence, EEMD was utilized to eliminate the influence of background noise and extract the first mode signal of the structure. IMF4 was selected to conduct CMSCE to evaluate the complexity between two signals. The template length *m*, threshold *r*, and signal length *N* were set to 4, 0.08 × SD of the time series, and 20,000 points, respectively. Similarly, the DI was calculated to time scale 10 (τ = 10). The damaged cases were compared with the undamaged case, and then the DI was applied to quantify the CMSCE results.

**Figure 12:**
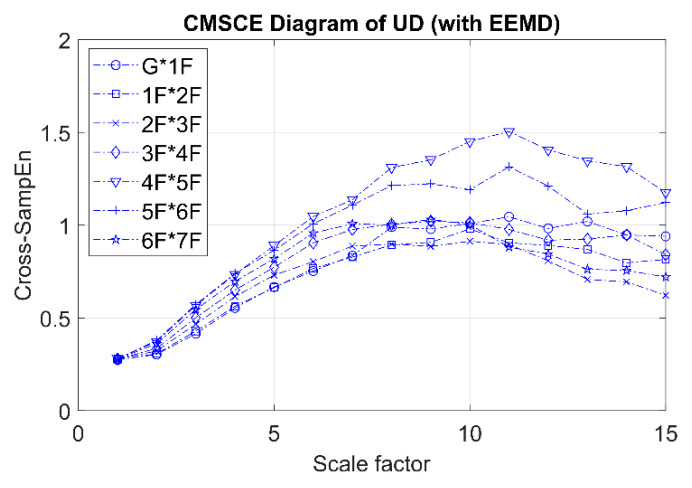
CMSCE diagram of undamaged case with EEMD.

Figure 12 presents the CMSCE diagram obtained through EEMD for the undamaged case. G*1F denotes the curve for the first floor and 1F*2F denotes the curve for the second floor; the curves for the remaining floors follow similar designations. The entropy gradually increased with the time scale and reached a plateau at scale 10, where information richness could be accumulated if the system responded well. Moreover, the complexity rankings did not follow the order of the floors from low to high. Complexity was fairly consistent, except for the curves for the fourth and fifth floors.

**Figure 13:**
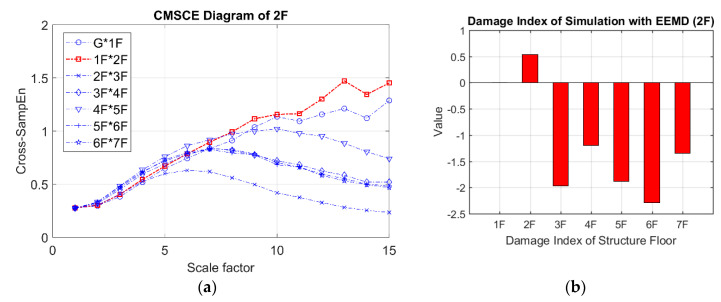
(**a**) CMSCE diagram of damage on the second floor (2F) with EEMD; (**b**) Damage index of damage on the second floor with EEMD.

Figure 13 presents the CMSCE diagram for damage on the second floor. The entropy values obtained for the second floor gradually increased to a peak at scale 10, as did the values for the first floor. All floors had almost identical complexity before time scale 5; the curves diverged between long scales. Regarding the DI results, a positive value for the second floor revealed that damage had occurred. Hence, the removal of bracings could result in significant differences among entropy curves.

**Figure 14:**
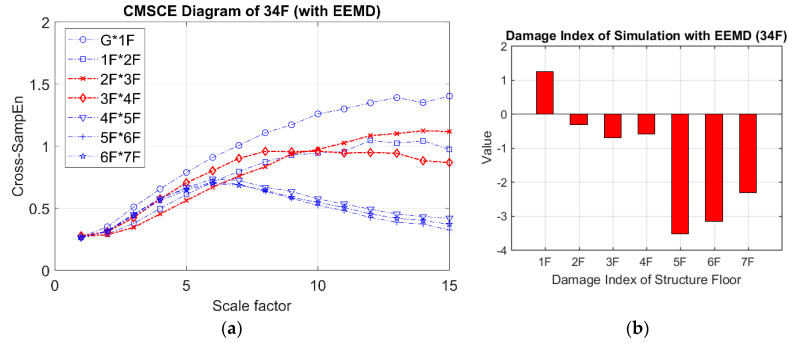
(**a**) CMSCE diagram of damage on the third and the fourth floor (34F) with EEMD; (**b**) Damage index of damage on the third and the fourth floor with EEMD.

**Figure 15:**
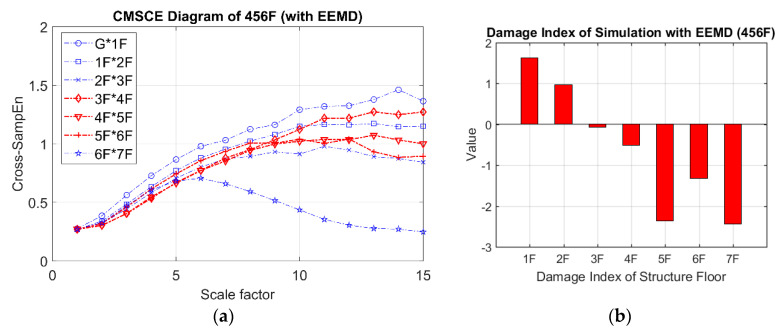
(**a**) CMSCE diagram of damage from the fourth to the sixth floor (456F) with EEMD; (**b**) Damage index of damage from the fourth to the sixth floor with EEMD.

The results of damage assessment on the third and fourth floors are presented in Figure 14. Compared with those for the damaged floors, the curves for the healthy floor above the damaged floor, the fifth to seventh floors, slowly descended after time scale 6. The curves for the third and fourth floors increased, but the increase was not evidently greater than that of the curves for the undamaged case, which caused a misclassification in the DI analysis. In addition, the same trend occurred in the case of three-story damage, as shown in Figure 15. Figure 15a shows the CMSCE diagram for damage from the fourth floor to the sixth floor, as observed through analysis with EEMD; the curve of the undamaged floor above the sixth floor was lower than those for the sixth floor. Moreover, the curves for the damaged floor sustained a stable plateau, indicating a high degree of complexity. Nevertheless, the first and second floors were misclassified as damaged in the DI analysis because this damage reduced the stability of the entire structure.

**Figure 16:**
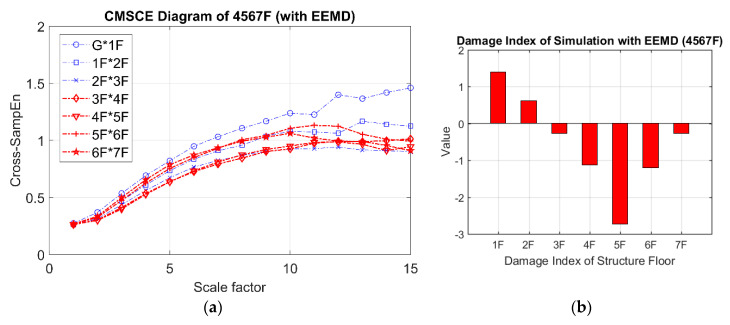
(**a**) CMSCE diagram of damage from the fourth to the seventh floor (4567F) with EEMD; (**b**) Damage index of damage from the fourth to the seventh floor with EEMD.

The CMSCE diagram for multistory damage is illustrated in Figure 16a. The curves for the fourth floor to the seventh floor gradually ascended and reached a plateau at scale 10. However, a comparison of the results in Figure 16a with those for the undamaged case (Figure 12) revealed that the overall entropy values were almost identical, signifying that the damage location could not be detected by applying the first mode signal. The obscure change in the CMSCE diagram resulted in a failure to diagnose the damaged floor in the DI results.

#### 3.3.3. Discussion on Numerical Simulation

A total of 15 damage cases were examined to verify the feasibility of CMSCE and evaluate the effectiveness of using EEMD to extract the structural first mode. A two-class statistical classification system, namely a confusion matrix, was applied for further performance evaluation. The entries in a confusion matrix are explained as follows: true positives (TP), referring to the number of “yes” predictions for “yes” instances; true negatives (TN), referring to the number of “no” predictions for “no” instances; false positives (FP), referring to the number of “yes” predictions for “no” instances; and false negatives (FN), referring to the number of “no” predictions for “yes” instances. On the basis of these entries, three indices can be calculated as follows:

(15) $$\mathrm{Accuracy}=\frac{\mathrm{TP}+\mathrm{TN}}{\mathrm{TP}+\mathrm{TN}+\mathrm{FP}+\mathrm{FN}}\;\mathrm{Precision}\;=\;\frac{\mathrm{TP}}{\mathrm{TP}\;+\;\mathrm{FP}}\;\mathrm{Recall}\;=\;\frac{\mathrm{TP}}{\mathrm{TP}\;+\;\mathrm{FN}}$$

In this study, on the basis of the definitions of the confusion matrix, the DI results were first classified into four categories: TP, indicating that the damaged floor had been correctly identified; TN, indicating that the undamaged floor had been correctly classified as healthy; FP, indicating that the healthy floor had been misclassified as damaged; and FN, indicating that the damaged floor had been misclassified as healthy. Subsequently, the three indices were calculated. The classification results are listed in Table 2. Accuracy denoted the overall accuracy of the classifier. Precision referred to the proportion of instances that had been classified as damaged and were actually damaged. “Recall” referred to the proportion of damaged instances that had been correctly classified as damaged. Hence, precision could be considered a measure of exactness, and recall could be considered a measure of completeness.

In the analysis without EEMD, a high accuracy rate of 93.9% was obtained, and a precision of 92% was obtained because two floors were misclassified as damaged. Subsequently, a recall rate of 85.2% was observed, revealing a high likelihood of detecting actual damage. By contrast, in the analysis with EEMD, the observed accuracy was 77.6% and precision was 66.7%, which was too low to precisely classify the healthy floor. Moreover, the observed recall was only 37% because the inconspicuous change in complexity resulted in errors in the detection of damaged floors. The method without EEMD was considered to be appropriate in monitoring structure.

#### 3.3.4. Noise Statistical Analysis

The CMSCE without EEMD has been numerically demonstrated to be an effective SHM method. However, the basic assumption of ambient vibration condition in the methodology may be changed in practical application. In order to verify the robustness of the proposed method, different levels of noise are randomly added into the original time series for noise statistical analysis. The noise is simulated by a Gaussian white-noise. The signal-to-noise ratio (SNR) values are chosen to be 60 dB, 40 dB, and 20 dB, respectively. The damage location is diagnosed by the CMSCE and damage index methods. The symbol C indicates that the damage can be correctly identified and F represents for false identification. As shown in the Table 3, the accuracy of the method based on CMSCE remains the same for the accuracy of 78.57% under all the cases of SNR 60, 40, and 20, which shows no influence by possible external noise. The performance of the damage index has the same result of 85.71% accuracy under SNR 60 and 40. For a higher noise level (SNR 20), the damage index drops slightly on damage location assessment and has the same result of 78.57% as the CMSCE method. The result has proven that a reliable result can be provided by the proposed methods when the ambient condition is affected by possible external noise.

## 4. Experimental Verification

### 4.1. Experimental Setup

**Figure 17:**
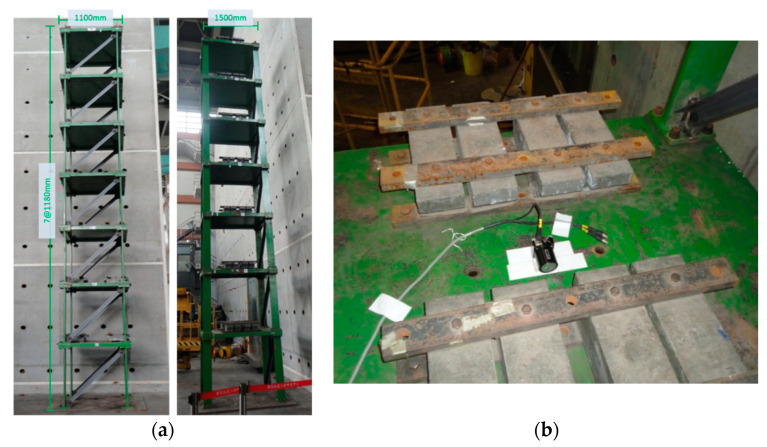
(**a**) A seven-story scale-down benchmark structure; (**b**) The arrangement of velocity meter and mass block.

To verify the practicality of the SHM system, an ambient vibration experiment for a scaled-down steel benchmark structure was conducted. The number of damaged cases was designed to be identical to that of the aforementioned numerical simulation. Moreover, the characteristics of the experimental structure were similar to those of the numerical model; the height of each floor was 1.1 m, and the widths of each floor were 1.5 and 1.1 m. An additional mass of 500 kg (Figure 17b) was added to each floor to simulate the actual structural characteristics. An L-shaped steel angle measuring 65 mm × 65 mm × 6 mm was selected as the bracing.

**Figure 18:**
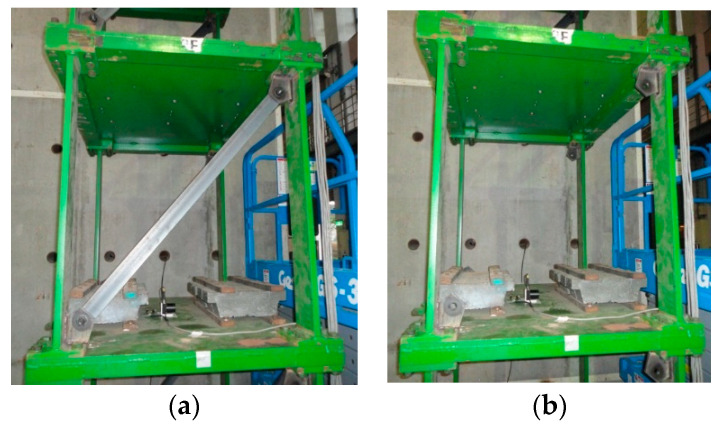
The damage simulation: (**a**) the healthy condition; (**b**) the damaged condition.

**Figure 19:**
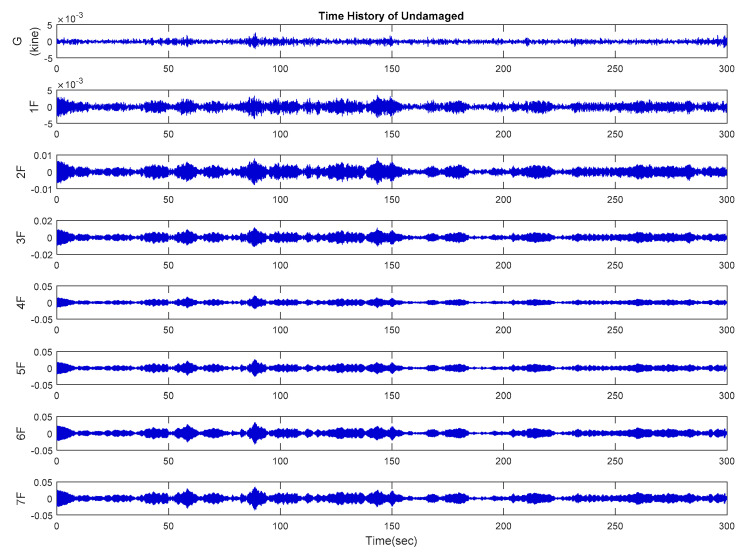
The velocity response of the undamaged case under ambient vibration.

For data acquisition, a sensitive velocity sensor VSE-15D (Tokyo Sokushin, Tokyo, Japan) was mounted on each floor to record the ambient vibration from the weak axis. Similar to the numerical analysis, damage was simulated as the removal of the bracing in the weak axis direction. The damage simulation is depicted in Figure 18. The experiment was executed at night to avoid interference from the testing field. Therefore, the velocity response under ambient vibration on each floor was recorded at a sampling rate of 200 Hz. A set of data was measured for 300 s, and then four sets were recorded to eliminate variance. The response signals of the undamaged case are shown in Figure 19.

The velocity responses of each case were examined using fast Fourier transform. Subsequently, the response of the seventh floor for each case was selected to examine the rationality of the signal; the results are listed in Table 4. The healthy condition had the highest frequency (3.34 Hz). Additionally, the frequency decreased markedly as damage occurred on the bottom floor, indicating that the removal of the bracing on the low floor had a relatively severe effect on the global stiffness of the structure.

### 4.2. Damage Detection Result

In all damage cases, the original velocity signals were analyzed to evaluate the complexity between floors. The parameters template length *m*, threshold *r*, and signal length *N* were optimized as 4, 0.08 SD of the time series, and 20,000 points, respectively. The DI range was identical to that of the simulation: from scale 1 to 10. In addition, low positive values were excluded by a predetermined threshold value 1 during the practical application [30]. Consequently, the damaged floor could be detected.

**Figure 20:**
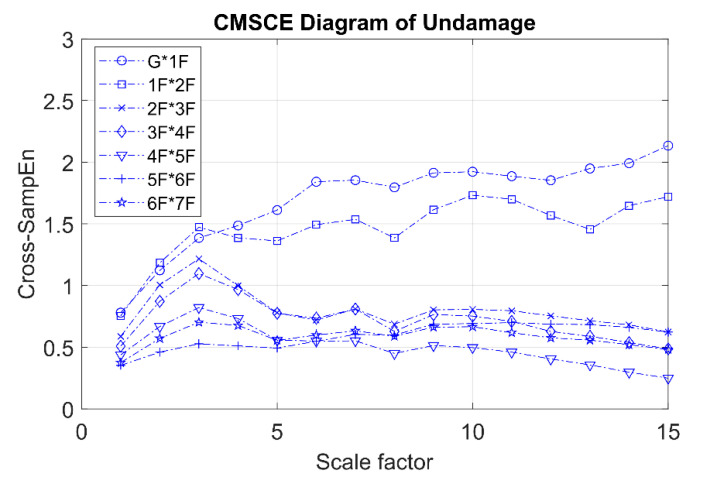
The experimental CMSCE diagram of undamaged case.

Figure 20 presents the CMSCE diagram for the healthy condition. The curve for the first floor was the highest on all scales, indicating high complexity between the ground and first floors. The curves for the third floor maintained a gap with those for the second floor and remained constant from scale 5 to 10; this reveals that the signals tended to be similar. The healthy condition was recognized as a reference for detecting damage locations.

**Figure 21:**
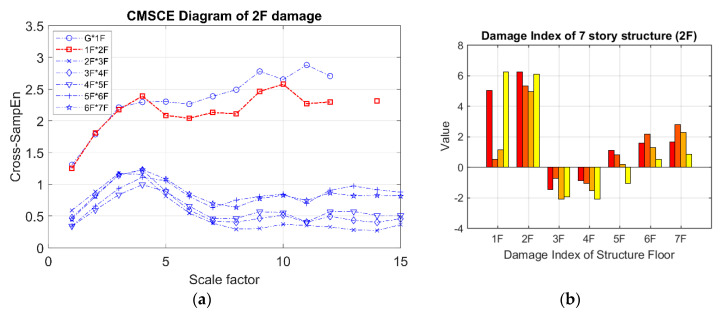
(**a**) The experimental CMSCE diagram of damage on the second floor (2F); (**b**) Damage index of damage on the second floor.

The CMSCE diagram for damage on the second floor is presented in Figure 21. The curve for the second floor increased evidently; however, the damage on the floor engendered an increase in the curve for the first floor. Even undefined entropies appeared from scale 12; the DI result was not influenced because the range was 1 to 10. According to the DI results, the index for the second floor was apparently higher than those for the other floors, whereas the first and fourth sets revealed an outlier on the first floor.

**Figure 22:**
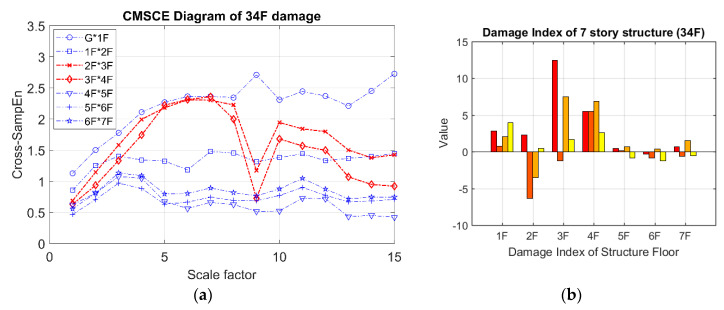
(**a**) The experimental CMSCE diagram of damage on the third and the fourth floor (34F); (**b**) Damage index of damage on the third and the fourth floor.

The CMSCE diagram for the two-story damaged case is illustrated in Figure 22a. The curves for damage on the third and fourth floors showed the highest complexity from scale 4 to 8. Compared with the curves for the undamaged case, the difference between the curves for the damaged floors was distinguishable. Moreover, the DI values for the third and fourth floors were positive, whereas the values for the other floors were close to zero. Therefore, the damage on the third and fourth floors could be detected.

**Figure 23:**
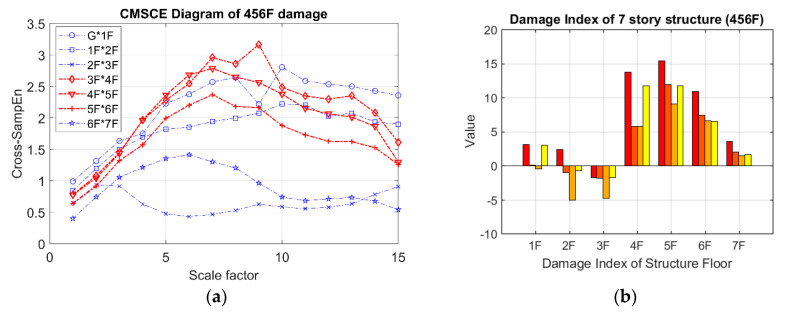
(**a**) The experimental CMSCE diagram of damage from the fourth to the sixth floor (456F); (**b**) Damage index of damage from the fourth to the sixth floor.

**Figure 24:**
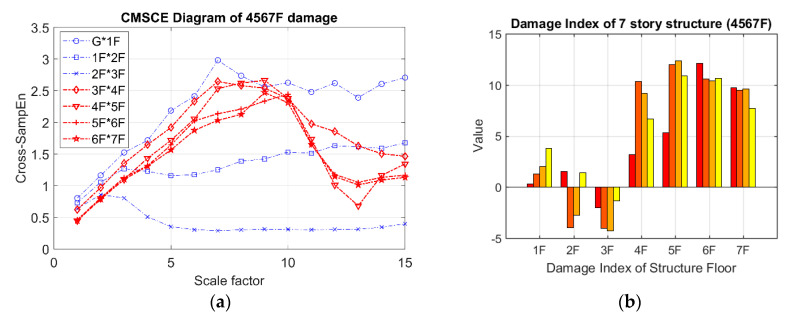
(**a**) The experimental CMSCE diagram of damage from the fourth to the seventh floor (4567F); (**b**) Damage index of damage from the fourth to the seventh floor.

Figure 23 shows the curves for three-story damaged case (i.e., damage from the fourth floor to the sixth floor). The damaged curves ascended rapidly at scale 5; however, the curve for the seventh floor slightly increased from scale 4 to 9 because of the loss of stiffness from the fourth floor to the sixth floor. The DI was used to quantify the CMSCE diagram, and the results showed that damage had occurred from the fourth floor to the seventh floor. A low positive value, which was excluded, could be observed for the seventh floor. Regarding the more severe damage condition, the results for damage from the fourth floor to the seventh floor are illustrated in Figure 24. A similar trend could be observed, revealing that the curves for damage increased rapidly at scale 4. In addition to the curves for damage, the curve for the first floor was elevated at scale 5 to 10, resulting in low positive DI values. The DI diagram is presented in Figure 24b. Apparently, the damage from the fourth floor to the seventh floor could be detected rapidly.

The confusion matrix was next utilized to evaluate the performance of the DI results. First, the results were classified into four categories: TP, TN, FP, and FN. As shown in Table 5, an accuracy rate of 78.6% was obtained. Also, a precision of 56.8% was observed, indicating that the method was too conservative to classify the healthy floor. The low accuracy and precision phenomenon may be caused by the unexpected noise condition or signal interference faced in practical measurement and can be improved by slightly adjusting the threshold value. Nevertheless, a strong recall rate of 92.6% was noted, revealing that most of the floors that had been diagnosed as damaged were correctly classified as damaged floors.

## 5. Conclusions

Although entropy analysis has been applied extensively in biomedicine, finance, and mechanical engineering, few studies have utilized entropy analysis in the field of SHM. Based on the previous studies, unstable entropy values are occasionally observed when multiscale cross-sample entropy (MSCE) is employed. Therefore, the CMSCE method was utilized to enhance the reliability of entropy values on every scale. A preliminary study on a three-story RC structure has demonstrated that the CMSCE method can largely improve the performance of the original MSCE-based SHM system. In addition, as most of the practical structural damage mainly causes a frequency change on the first fundamental mode, the effectiveness of extracting first mode signals by using EEMD was also attempted. The feasibility of detecting damage locations was verified in a numerical model. It is shown that the CMSCE without EEMD method has the best overall accuracy for different levels of damage. In general, 93.9% of the floors were correctly classified and 85.2% of the actual damaged floors were correctly diagnosed. Moreover, the robustness of the SHM system was also verified through noise statistical analysis.

The proposed entropy-based SHM system for long-term monitoring was then tested by a series of experimental verification. The reliability and viability of the proposed SHM system were examined using 15 damaged cases in 5 categories representing several degrees of damage severity. The results obtained by observing the scaled-down benchmark structure reveal that 78.6% of the floors were correctly classified and 92.6% of the actual damaged floors were correctly diagnosed. Based on the outcome obtained from both numerical simulation and experimental verification, two advantages of applying CMSCE including the enhancement of system reliability on noise interference and the improvement of diagnosis accuracy have been achieved. The proposed entropy-based SHM system has been demonstrated to have high potential for practical application.

## Figures and Tables

**Table 1:** Damage cases and modal analysis.

Case Number	Damage Case	Frequency (Hz)	
		SAP2000	IMF4
1	Undamaged	3.16	3.12
2	1F	2.56	2.49
3	2F	2.55	2.49
4	3F	2.63	2.68
5	4F	2.73	2.73
6	5F	2.85	2.88
7	6F	2.99	2.98
8	7F	3.12	3.12
9	1&2F	2.15	2.15
10	3&4F	2.32	2.34
11	5&6F	2.69	2.69
12	1&2&3F	1.93	1.95
13	4&5&6F	2.38	2.34
14	1&2&3&4F	1.81	1.81
15	4&5&6&7F	2.35	2.34

**Table 2:** Results of confusion matrix for numerical model.

Two-Class Statistical Classification: Confusion Matrix									
Case Number	Damage Floors	CMSCE				EEMD + CMSCE			
		TP	FP	TN	FN	TP	FP	TN	FN
1	None								
2	1F	0	0	6	1	1	0	6	0
3	2F	1	0	6	0	1	0	6	0
4	3F	1	0	6	0	1	0	6	0
5	4F	1	0	6	0	1	0	6	0
6	5F	1	0	6	0	0	0	6	1
7	6F	1	0	6	0	0	0	6	1
8	7F	1	1	5	0	1	0	6	0
9	1&2F	1	0	5	1	1	0	5	1
10	3&4F	2	0	5	0	0	1	4	2
11	5&6F	2	0	5	0	0	0	5	2
12	1&2&3F	2	1	3	1	1	0	4	2
13	4&5&6F	3	0	4	0	0	2	2	3
14	1&2&3&4F	3	0	3	1	3	0	3	1
15	4&5&6&7F	4	0	3	0	0	2	1	4
Total		23	2	69	4	10	5	66	17
Accuracy		93.9%				77.6%			
Precision		92%				66.7%			
Recall		85.2%				37%			

**Table 3:** The accuracy of CMSCE and damage index method for different noise levels.

Damage Location	SNR = 60		SNR = 40		SNR = 20	
	CMSCE	Damage Index	CMSCE	Damage Index	CMSCE	Damage Index
1F	C	F	C	F	C	F
2F	C	C	C	C	C	C
3F	C	C	C	C	C	C
4F	C	C	C	C	C	C
5F	C	C	C	C	C	C
6F	F	C	F	C	F	C
7F	F	C	F	C	F	F
1&2F	C	C	C	C	C	C
3&4F	C	C	C	C	C	C
5&6F	F	C	F	C	F	C
1&2&3F	C	C	C	C	C	C
4&5&6F	C	F	C	F	C	F
1&2&3&4F	C	C	C	C	C	C
4&5&6&7F	C	C	C	C	C	C
Accuracy (%)	78.57%	85.71%	78.57%	85.71%	78.57%	78.57%

Note: C = Correct, F = False.

**Table 4:** FFT results of the experimental data.

Case Number	Damage Group	Damage Floors	Frequency (Hz)
1	Undamaged	None	3.34
2	One-story damage	1F	2.08
3		2F	2.13
4		3F	2.12
5		4F	2.29
6		5F	2.61
7		6F	2.88
8		7F	3.2
9	Two-story damage	1&2F	1.64
10		3&4F	1.83
11		5&6F	2.32
12	Three-story damage	1&2&3F	1.44
13		4&5&6F	1.88
14	Multistory damage	1&2&3&4F	1.33
15		4&5&6&7F	1.86

**Table 5:** Results of the confusion matrix for experimental verification.

Two-Class Statistical Classification: Confusion Matrix					
Case Number	Damage Floors	CMSCE			
		TP	FP	TN	FN
1	None				
2	1F	1	0	6	0
3	2F	1	3	3	0
4	3F	1	4	2	0
5	4F	1	3	3	0
6	5F	1	0	6	0
7	6F	1	0	6	0
8	7F	0	1	5	1
9	1&2F	2	2	3	0
10	3&4F	2	1	4	0
11	5&6F	2	1	4	0
12	1&2&3F	2	0	4	1
13	4&5&6F	3	1	3	0
14	1&2&3&4F	4	3	0	0
15	4&5&6&7F	4	0	3	0
Total		25	19	52	2
Accuracy		78.6%			
Precision		56.8%			
Recall		92.6%			
